# Recent Advances in Parkinson’s Disease Research: From Pathophysiology to Novel Therapeutic Approaches

**DOI:** 10.3390/biomedicines13092283

**Published:** 2025-09-17

**Authors:** Sujung Yeo

**Affiliations:** College of Korean Medicine, Sang Ji University, Wonju 26339, Republic of Korea; pinkteeth@naver.com

## 1. Introduction

Parkinson’s disease is a progressive neurodegenerative disorder affecting approximately 1–2% of the population over 65 years of age [1]. The cardinal pathological hallmarks include selective loss of dopaminergic neurons in the substantia nigra pars compacta [2], formation of Lewy bodies containing misfolded α-synuclein protein [3], and consequent disruption of the nigrostriatal dopaminergic pathway [4]. The clinical manifestations encompass motor symptoms (bradykinesia, rigidity, tremor, and postural instability) [5] and non-motor features (cognitive dysfunction, autonomic dysfunction, and sleep disorders) [6]. Understanding the multifaceted pathophysiological mechanisms underlying PD is crucial for developing disease-modifying therapeutics and improving clinical outcomes.

For the Special Issue “Parkinson’s Disease: From Pathophysiology to Novel Therapeutic Approaches,” a total of 10 papers (9 articles and 1 review) underwent a rigorous peer review process. These studies focused on molecular and genetic mechanisms, neuroprotective therapeutic strategies, and diagnostic innovations from a neuroscientific perspective.

## 2. Molecular Genetic Mechanisms and Cellular Pathways

Recent investigations have significantly advanced our understanding of the genetic architecture and molecular pathophysiology of PD, revealing novel therapeutic targets and mechanistic insights.

### 2.1. Genetic Heterogeneity and Population-Specific Variants

Salemi et al. (2023) investigated genetic variations in a Sicilian cohort of PD patients using next-generation sequencing (NGS) technology. The research team analyzed DNA from 126 patients using a customized gene panel encompassing 162 genes. The most frequent variants were identified in the LRRK2, GBA, POLG, and PRKN genes, with some variants not previously associated with PD. This study highlights that advancing genetic understanding may enhance diagnostic accuracy and support the development of novel therapeutic strategies.

The impact of specific genetic variants on treatment response was further elucidated by Bispo et al. (2023), who investigated the association between PRKN gene mutations [7] and levodopa-induced dyskinesia (LID) [8]. LID is a debilitating complication affecting approximately 40–50% of PD patients after 5 years of levodopa therapy [9]. Patients who developed dyskinesia generally had younger age at symptom onset, longer duration of PD, extended treatment duration, and received higher levodopa equivalent daily dose. However, no statistically significant association was found between the investigated *PRKN* variants and LID occurrence.

### 2.2. Mitochondrial Dysfunction and Neuroinflammatory Cascades

Guedes et al. (2023) reviewed the role of microRNAs (miRNAs) in PD pathogenesis, emphasizing their involvement in key pathogenic mechanisms including mitochondrial dysfunction and immune activation. The study also highlighted the gut–brain axis, discussing how alterations in gut microbiota may interact with miRNAs as potential biomarkers in PD development. The authors propose miRNAs as potential biomarkers and therapeutic targets for novel disease-modifying strategies [10].

### 2.3. α-Synuclein Proteostasis and Aggregation Pathways

Seo and Yeo (2023) investigated the effects of Serping1 siRNA on α-synuclein regulation in an MPTP-induced mouse model of PD. They demonstrated that Serping1 siRNA reduced both Serping1 and α-synuclein expression in the colon while alleviating α-synuclein aggregation in the brain. These findings suggest Serping1 as a potential therapeutic target for PD, although the molecular mechanisms underlying its regulation of α-syn aggregation require further clarification.

## 3. Neuroprotective Strategies and Therapeutic Innovations

Contemporary research has focused on identifying and validating neuroprotective compounds and novel therapeutic modalities that can halt or slow disease progression while ameliorating clinical symptoms.

### 3.1. Natural Compound-Based Neuroprotection

The neuroprotective potential of natural compounds has garnered significant attention due to their multi-target mechanisms and favorable safety profiles.

Quercetin Nanocrystals: Lakshmi et al. (2023) explored the therapeutic potential of quercetin nanocrystals (QNCs) using network pharmacology and in vivo models. QNCs demonstrated modulation of dopamine receptors D2 and D4, which are critical in PD progression [11]. In mice, QNCs improved motor deficits, reduced oxidative stress, restored dopamine levels, and attenuated neuronal damage more effectively than standard quercetin. These findings highlight QNCs as a promising candidate with enhanced bioavailability for PD therapy.

Licochalcone D: Oh et al. (2023) examined the neuroprotective effects of licochalcone D (LCD) in primitive neural stem cells (pNSCs) derived from patient-specific induced pluripotent stem cells (iPSCs). Under MG132-induced oxidative stress, LCD reduced cell death through modulation of the EGFR/AKT and JNK signaling pathways. Mechanistically, LCD directly bound to JunD, regulating its expression, and thus suggesting LCD as a potential antioxidant agent for preventing PD-related pathological phenotypes [12].

Nicotinamide: Rehman et al. (2022) investigated nicotinamide (NAM) neuroprotective effects in an MPTP-induced mouse model. NAM treatment alleviated motor dysfunction, reduced α-synuclein accumulation, and restored tyrosine hydroxylase and dopamine transporter levels. Mechanistically, NAM enhanced Nrf2/HO-1 signaling to counteract oxidative stress while suppressing TLR4, phosphorylated NF-κB, and COX-2 to mitigate neuroinflammation.

Medicinal Mushroom Combinations: Cordaro et al. (2022) investigated the synergistic effects of *Hericium erinaceus* and *Coriolus versicolor* in modulating neuroinflammation and oxidative stress. Using a rotenone-induced mouse model, oral administration of these compounds, alone or in combination, reduced NF-κB–mediated inflammatory signaling and enhanced Nrf2-related antioxidant responses. These effects prevented dopaminergic neuronal loss and α-synuclein accumulation while alleviating both motor and non-motor deficits.

### 3.2. Precision Radiosurgical Interventions

Goc et al. (2023) reported Phase II clinical trial results evaluating frameless CyberKnife-based stereotactic radiosurgery for tremor management in PD patients. Twenty-three patients received single-fraction doses ranging from 70 to 105 Gy. The findings demonstrated meaningful tremor control in most patients, with doses below 90 Gy associated with fewer adverse effects. This approach may serve as an alternative for patients ineligible for deep brain stimulation [13].

## 4. Metabolomic Biomarker Discovery

Gatarek et al. (2022) conducted comprehensive metabolomic profiling of plasma samples from PD patients to identify potential biomarkers and reveal disease-related metabolic pathways. The study identified abnormal metabolic changes related to amino acid metabolism, TCA cycle metabolism, and mitochondrial function in PD patient plasma, providing opportunities for further research into pathogenesis, progression monitoring, and therapeutic efficacy evaluation.

## 5. Future Directions and Clinical Translation

Recent advances in PD research demonstrate remarkable progress in understanding disease mechanisms, developing therapeutic strategies, and improving diagnostic capabilities. Several key areas warrant particular attention.

### 5.1. Precision Medicine Implementation

The integration of genetic profiling, biomarker assessment, and personalized treatment algorithms represents a paradigm shift toward precision medicine in PD management. Future studies should focus on developing comprehensive diagnostic panels incorporating genetic variants, protein biomarkers, and metabolomics to guide individualized treatment decisions.

### 5.2. Disease-Modifying Therapeutic Development

The translation of promising preclinical findings into clinical applications requires rigorous validation through well-designed clinical trials. Particular emphasis should be placed on developing combination therapies that target multiple pathophysiological pathways simultaneously, potentially offering synergistic neuroprotective effects.

### 5.3. Early Detection and Intervention

Identifying prodromal biomarkers and developing interventions for at-risk individuals represent critical research areas. Early therapeutic intervention during the preclinical phase may offer the greatest potential for disease modification and improved long-term outcomes.

## 6. Conclusions

Recent research demonstrates significant advances in understanding PD pathophysiology and therapeutic development. Key findings highlight complex interactions between genetic factors, mitochondrial dysfunction, protein aggregation, and neuroinflammation at unprecedented molecular detail.

The exploration of natural neuroprotective compounds and innovative therapies like precision radiosurgery shows promise for clinical application. Biomarker discovery through metabolomics and improved diagnostic methods are advancing early detection and intervention capabilities.

Moving forward, the field is progressing toward personalized medicine by integrating multi-omics data with clinical phenotyping. Future priorities include translating mechanistic insights into effective treatments, developing robust diagnostic biomarker panels, and ultimately creating disease-modifying therapies that can transform PD from a progressive disorder into a manageable condition.
